# Cochlear implant electrode sealing techniques and related intracochlear pressure changes

**DOI:** 10.1186/s40463-017-0218-y

**Published:** 2017-05-11

**Authors:** Ingo Todt, Julica Utca, Dania Karimi, Arne Ernst, Philipp Mittmann

**Affiliations:** Department of Otolaryngology, Head and Neck Surgery, Unfallkrankenhaus Berlin, Warenerstr.7, 12683 Berlin, Germany

**Keywords:** Cochlea implant, Round window, Sealing, Intracochlear pressure

## Abstract

**Background:**

The inserted cochlear implanted electrode is covered at the site of the round window or cochleostomy to prevent infections and leakage. In a surgically hearing preservational concept, low intracochlear pressure changes are of high importance. The aim of this study was to observe intracochlear pressure changes due to different sealing techniques in a cochlear model.

**Methods:**

Cochlear implant electrode insertions were performed in an artifical cochlear model and the intracochlear pressure changes were recorded in parallel with a micro-pressure sensor positioned in the apical region of the cochlea model to follow the maximum amplitude of intracochlear pressure. Four different sealing conditions were compared: 1) overlay, 2) overlay with fascia pushed in, 3) donut-like fascia ring, 4) donut-like fascia ring pushed in.

**Results:**

We found statistically significant differences in the occurrence of maximum amplitude of intracochlear pressure peak changes related to sealing procedure comparing the different techniques. While the lowest amplitude changes could be observed for the overlay technique (0.14 mmHg ± 0.06) the highest values could be observed for the donut-like pushed in technique (1.79 mmHg ± 0.69).

**Conclusion:**

Sealing the electrode inserted cochlea can lead to significant intracochlear pressure changes. Pushing in of the sealing tissue cannot be recommended.

## Background

Cochlear implantation (CI) is a globally accepted treatment for children and adults with severe-to-profound hearing loss. In recent years, the indications for cochlear implantation have been widened to patients with substantial residual hearing. To avoid complications such as perilymphatic leakage, the loss of residual hearing, vertigo and ascending infections,tight sealing of the cochleostomy or the round window membrane is an important goal for CI surgeons. On the other hand, it has been shown that intracochlear pressure (ICP) changes occur during the implantationprocedure; these are relevant factors in terms of hearing preservation shown clinically and underlined experimentally. ICP changes in a model have been described which correlate to the insertion speed [1] of a cochlear implant electrode insertion. Different forms of opening an artificial round window have been shown to cause significant differences in ICP changes [2, 3], as well as the size of the round window opening and the hydrophilised state of the cochlear implant electrode [4] and post-insertional cable movements [5]. Clinically it has been shown that speed of insertion [6], underwater insertion [7] and the size of the round window opening and moisturisation of the electrode [8] are important factors for hearing preservation.

The aim of the present studywas to investigate the effect of different methods of electrode sealing on the ICP in a model cochlea.

## Methods

### Model and sealing techniques

#### Pressure sensor

The ICP was measured using a micro-optical pressure sensor 0,8 mm FOP (FISO, Canada). Basically, the tip of the pressure sensor is a hollow glass tube sealed on one end by a plastic thin film diaphragm coated with a reflective surface of evaporated gold. The optical fiber is located in the glass tube with a small distance (50–100 μm) to the diaphragm tip. The optical fiber is attached to a LED light source and to a photodiode sensor. Light from the LED source reaches the sensor tip of the optical fiber, fans out as it exits the fiber and is reflected by the gold-covered flexible diaphragm. The reflected light is sensed by the photodiode. Small amounts of pressure induced distance displacements of the diaphragm, which modulate the intensity of reflected light. The sensor is connected with a module, which is again linked to a computer. Evolution software was used to record the ICP. The time sensitivity of the sensor was 300 measurements per second. Low pass filter was set to 500Hz.

### Model

**Fig. 1:**
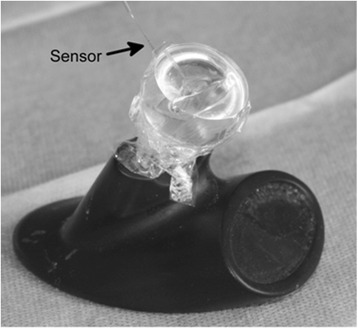
Cochlear model for pressure experiments

The model was a full-scale model of the cochlea, distributed by Advanced Bionics and MedEl for surgical training with a volume of 87 mm^3^ (Fig. 1), which is slightly above the physiological range [9]. The sensor was positioned through a drilled hole in the apical region of the cochlea. The sensor was fixed in its position with fibrin glue and placed within the channel in such a way that the tip was not in contact with the edge of the channel or the ground. Afterwards, the cochlea was microscopically controlled to exclude any enclosed air bubbles. The experiments were in series with a sensor in an unchanged position to exclude sensor position-related bias and to allow inter-experimental comparability. All procedures were performed with a High Focus midscalar electrode (Advanced Bionics, Stäfa, Swiss).

### Analysis

Statistically, the maximum amplitude of pressure change was calculatedand statistically analysed by an independent *t*-test (SPSS 10.00). This study was approved by the institutional review board (*IRB-ukb-HNO-2015/10*)

### Experiments

1. Overlay sealing:
  The artifical RW opening beside the inserted electrode was covered by a strip of fat. All experiments were performed five times.
2. Overlay sealing with push in:
  The artifical RW opening beside the inserted electrode was covered by a strip of fat. The fat was pushed between the RW edge and electrode. All experiments were performed five times.
3. Donut-like sealing:
  A perforated piece of fat was created, in whichan electrode was inserted. This donut-like seal was inserted into the artifical RW until it was closed. All experiments were performed five times.
4. Donut-like seal pushed in:
  A perforated piece of fat was created, in whichan electrode was inserted. The electrode was inserted and the donut-like seal was pushed down the electrode until the RW was closed. All experiments were performed five times.

## Results

**Fig. 2:**
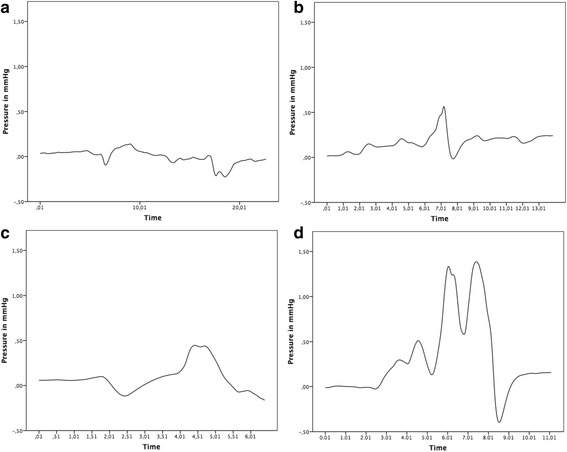
**a** Exemplaric pressure change related to an overlay sealing. **b** Exemplaric pressure change related to an overlay push in sealing. **c** Exemplaric pressure change related to a donut-like sealing. **d** Exemplaric pressure change related to a donut-like push in sealing

A one-way ANOVA was conducted to determine whether the mean maximum ICP (mmHg) was different between the variable sealing techniques. Data are presented as mean ± standard deviation. The mean maximum ICP increased from overlay (1) (0.14 ± 0.06), to donut like (3) (0.44 ± 0.27), to overlay pushed in (2) (0.56 ± 0.3) to donut like push in (1.79 ± 0.69) in that order (Fig. 2).

**Fig. 3:**
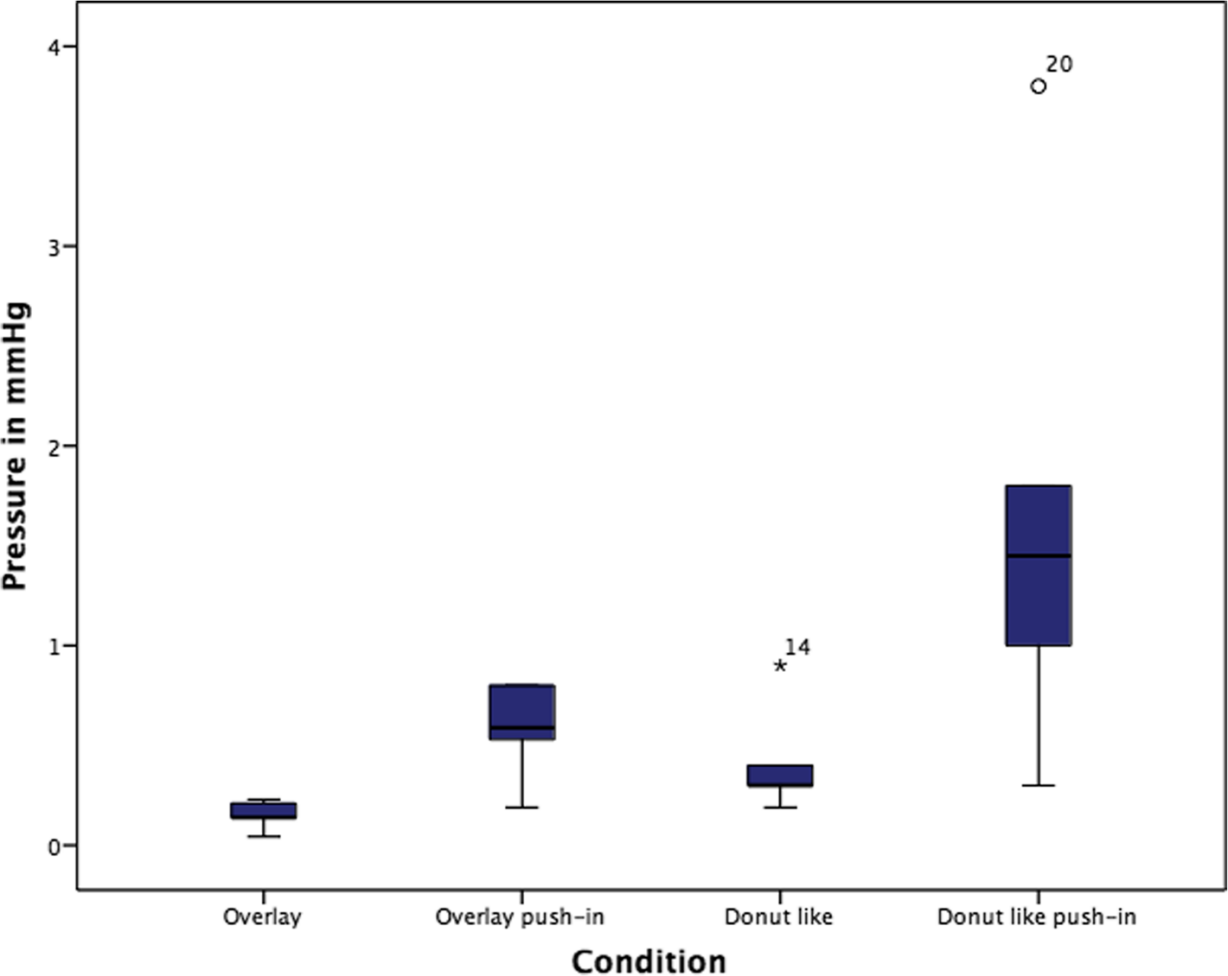
Comparison of intracochlear pressure changes related to different sealing techniques

The differences between these techniques were statistically significant (F(3, 16) = 16.615, *p* < 0.001). The data were normally distributed for each group, asassessed by a Shapiro-Wilks test (*p* < 0.05). Homogeneity of variances was violated, as assessed by Levene’s Test of Homogeneity of Variance (*p* = 0.003). Games-Howell post hoc analysis revealed that the difference from donut-like push in (4) to overlay (1) (1.65, 95% CI (0.4 to 2.9)) was statistically significant (*p* = 0.019), as well as from donut- like push in (4) to overlay push in (2) (1.23, 95% CI (0.04 to 2.43), *p* = 0.045) and from donut-like push in (4) to donut-like (3) (1.36, 95% CI (0.16 to 2.56), *p* = 0.031) (Fig. 3).

## Discussion

The sealing of the cochlear implant electrode is so far mostly observed under the aspect of tightness of the seal and a possible interaction of the sealing tissue to induce local fibrosis [10–12]. Our observation focussed on a possible role of the procedure as cause for potentially pathophysiologicaI ICP changes.

Pathophysiologically relevant acoustic levels are assumed to lead to high static ICP change or fast pressure changes with a high angular speed [13, 14]. Experimentally different aspects of the pre-, intra- and postinsertional procedures have been shown to to significantly affect ICP like round window opening [2–4], moisturizing the electrode [4], stabilization of the insertional hand [15], speed of insertion [1], electrode design [16, 17] and postinsertional cable movement [5]. Recent clinical studies underline ICP as an important factor [7, 8].

The packing of a cochlear implant electrode to seal the cochlea led anecdotally to a decrease of the intraoperative EcochG threshold and has an effect on basal ECAP thresholds [18].

This observation led to the question of a possible impact of the sealing procedure on the ICP, which possibly contributes to a decrease of residual hearing.

An impact of the sealing handling of the electrode on the ICP is likely since the seal separates the fluid filled cochlea from the aerated middle ear. By that, every handling is transmitted into the cochlea.

Our observations showed that as long as it is manually attempted to close the local leak, by covering it, pressure remains at a low level (Fig. 2). By trying to further increase the tightness of the seal by a push in or by optimising the circumferential covering, the pressure increases significantly (Fig. 3). The circumferential covering has the effect that movements of the electrode are transmitted into the cochlea like a cylinder stroke in a machine by inducing a sucking and pushing of fluid.

The pressure increase in terms of absolute volume is comparable to a sound pressure equivalent of 130 dB.

The transfer of the observation to the in vivo situation is limited in terms of two main points. The visibility and area to manipulate in vivo is worse related to the limited space through the posterior tympanotomy. This makes a tight circumferential sealing more difficult, as in the experimental situation. Secondly, manipulation around the electrode to reach a tight seal is less likely to be reachable in the in vivo situation, and the amount of handling in terms of touching and moving the electrode should be more extensive in vivo. Another point is the used HighFocus MS electrode. It differs from other electrodes by its basal diameter. It can be assumed that in smaller electrodes (e.g., Cochlear slim straight) and larger electrodes (e.g., Medel Flex series) the handling is different and therefore the occurence of ICP is different, too.

Based on our findings, pushing in of a seal should be avoided. A significant difference between a pure overlay of the donut-like technique could not be observed in terms of the generation of pressure. Surgically, not only the aspect of pressure generation and transmission into the cochlea has to be considered. In particular, perilymphatic leakage can be assumed to play a role in hearing preservation. Weakness of the study is the performance of the experiments in a cochlea model. Therefore natural pressure equilibration pathways (e.g., aqueductus cochleae, round window) are not considered in the pressure pattern.

Further studies focussing on the short- and long-term behaviour of seals seems to be of central importance to help to understand the role of the sealing in a hearing preservation concept.

## Conclusion

Sealing the inserted cochlea can lead to significant intracochlear pressure changes. Pushing in of the sealing tissue cannot be recommended.

## Abbreviations

CI: Cochlear implant
EcochG: Electrocochleography
ICP: Intracochlear pressure
RW: Round window
